# Psychometric Evidence on the Tampa Scale for Kinesiophobia for Temporomandibular Disorders: A Scoping Review

**DOI:** 10.3390/dj14080508

**Published:** 2026-08-11

**Authors:** Manuela Lalu, Marius Sorin Pop, Dana Carmen Zaha

**Affiliations:** 1 Doctoral School of Biomedical Sciences, University of Oradea, 410087 Oradea, Romania; 2 Pelican Hospital, 410469 Oradea, Romania; sorin.pop@spitalpelican.ro; 3 Department of Preclinical Discipline, Faculty of Medicine and Pharmacy, University of Oradea, 410087 Oradea, Romania; dzaha@uoradea.ro

**Keywords:** temporomandibular disorders, kinesiophobia, TSK-TMD, Tampa Scale for Kinesiophobia, psychometric properties

## Abstract

Temporomandibular disorders are frequently associated with orofacial pain, functional limitations, and psychosocial factors that may contribute to persistent symptoms. Kinesiophobia, or fear of movement, may influence mandibular function and rehabilitation outcomes. This scoping review aimed to map the available evidence on the psychometric properties and cross-cultural adaptations of the Tampa Scale for Kinesiophobia for Temporomandibular Disorders (TSK-TMD) and to identify gaps in the current literature. Literature searches were conducted in PubMed, Scopus, and the Web of Science Core Collection in March 2026. Reference lists of the included studies were also manually screened. English-language peer-reviewed studies reporting at least one measurement property of the TSK-TMD were included. The methodological quality of the included studies was assessed using the COSMIN Risk of Bias checklist for PROMs by applying the relevant measurement-property boxes and the “worst score counts” principle. The database searches identified 117 records; after removing 56 duplicates, 61 records were screened. Five full-text articles identified through database searching and one additional article identified through manual reference screening met the eligibility criteria, resulting in six included studies. The TSK-TMD appears to be a promising condition-specific patient-reported outcome measure, although measurement error, responsiveness, minimal important change, interpretability, and cross-cultural measurement invariance remain insufficiently investigated.

## 1. Introduction

Temporomandibular disorders (TMDs) represent a heterogeneous group of musculoskeletal and neuromuscular conditions affecting the temporomandibular joint, the masticatory muscles, and associated structures [1,2]. These disorders are frequently encountered in clinical practice and may lead to limitations in mandibular function, reduced quality of life, and functional disability [3,4]. Although symptoms may be self-limiting in some individuals, a considerable proportion of patients experience persistent pain and functional impairment, highlighting the need for comprehensive assessment and management approaches that consider both physical and psychosocial factors [5,6,7].

In recent years, increasing attention has been given to the role of psychological and behavioral factors in the development and persistence of chronic pain. Within the fear-avoidance model, fear of movement and re-injury, commonly referred to as kinesiophobia, may contribute to activity avoidance, physical deconditioning, hypervigilance, and pain-related disability [8,9,10]. In patients with TMD, kinesiophobia may influence mandibular movements and daily functional activities such as chewing, speaking, yawning, or engaging in therapeutic exercises [11,12]. As a result, the assessment of kinesiophobia may provide clinically relevant information beyond pain intensity or physical impairment alone [13].

The Tampa Scale for Kinesiophobia (TSK) was originally introduced by Miller et al. as a measure of fear of movement and reinjury and has since become one of the most widely used patient-reported outcome measures for assessing fear of movement in individuals with pain-related conditions [13,14]. To improve its relevance for patients with temporomandibular disorders, a condition-specific version, the Tampa Scale for Kinesiophobia for Temporomandibular Disorders (TSK-TMD), was developed [15]. Since its development, the TSK-TMD has been translated and culturally adapted into several languages, and validation studies have investigated its measurement properties, including internal consistency, test–retest reliability, structural validity, and construct validity (including convergent validity) [15,16,17,18,19,20].

However, the available evidence appears to be dispersed across different cultural contexts, populations, and validation studies. Some measurement properties have been evaluated more consistently than others, while aspects such as responsiveness, measurement error, interpretability, and cross-cultural measurement invariance remain less clearly established. Therefore, a scoping review is useful to map the current state of evidence, describe the extent and characteristics of existing research, and identify gaps that should be addressed in future validation studies.

The aim of this scoping review was to identify, map, and summarize the available literature on the Tampa Scale for Kinesiophobia for Temporomandibular Disorders, with particular focus on its cross-cultural adaptations and reported measurement properties, including structural validity, internal consistency, test–retest reliability, and construct validity (including convergent validity). A secondary aim was to identify areas where further psychometric investigation is needed.

## 2. Materials and Methods

### 2.1. Study Design

This scoping review was conducted to map the available evidence on the psychometric properties and cross-cultural adaptations of the Tampa Scale for Kinesiophobia for Temporomandibular Disorders (TSK-TMD). The review was guided by the Joanna Briggs Institute methodology for scoping reviews and reported in accordance with the Preferred Reporting Items for Systematic Reviews and Meta-Analyses extension for Scoping Reviews (PRISMA-ScR-checklist) [21,22]. A protocol was not registered for this scoping review.

A scoping review design was considered appropriate because the objective was to identify the extent, characteristics, and gaps of the available literature on the TSK-TMD, rather than to answer a narrowly focused effectiveness question or perform a quantitative synthesis.

### 2.2. Review Question

The main review question was:

What evidence is available regarding the psychometric properties and cross-cultural adaptations of the Tampa Scale for Kinesiophobia for Temporomandibular Disorders?

The secondary review questions were:

1. In which populations and cultural contexts has the TSK-TMD been evaluated?
2. Which measurement properties of the TSK-TMD have been investigated?
3. What gaps remain in the current evidence regarding the psychometric evaluation of the TSK-TMD?

### 2.3. Eligibility Criteria

Eligibility criteria were structured according to the Population–Concept–Context framework [21].

*Population*

Studies were considered eligible if they included individuals with temporomandibular disorders, temporomandibular symptoms, or populations in which the TSK-TMD was evaluated in relation to temporomandibular function, orofacial pain, or mandibular movement-related fear. Clinical TMD populations, mixed samples, and non-clinical populations were considered eligible if the study evaluated the TSK-TMD or one of its translated and culturally adapted versions.

*Concept*

The concept of interest was the Tampa Scale for Kinesiophobia for Temporomandibular Disorders, including the original version and translated or culturally adapted versions. Studies were included if they reported at least one measurement property of the TSK-TMD, such as internal consistency, test–retest reliability, structural validity, construct validity (including convergent validity), measurement error, responsiveness, interpretability, or cross-cultural validity.

*Context*

Studies from any geographical or cultural context were considered eligible. Only full-text articles published in English in peer-reviewed journals were included. No restrictions were applied regarding year of publication.

### 2.4. Inclusion and Exclusion Criteria

Studies were included if they:

- evaluated the Tampa Scale for Kinesiophobia for Temporomandibular Disorders or one of its translated/culturally adapted versions;
- reported at least one psychometric or measurement property of the instrument;
- included participants with temporomandibular disorders, temporomandibular symptoms, or a relevant population in which the TSK-TMD was psychometrically assessed;
- were published as full-text articles in peer-reviewed journals;
- were available in English.

Studies were excluded if they:

- evaluated only the general Tampa Scale for Kinesiophobia without adaptation for temporomandibular disorders;
- did not report any psychometric or measurement property of the TSK-TMD;
- were review articles, editorials, letters, conference abstracts, case reports, or opinion papers;
- were not available in English;
- did not provide sufficient information regarding the TSK-TMD.

### 2.5. Information Sources and Search Strategy

A comprehensive literature search was conducted in PubMed, Scopus, and the Web of Science Core Collection to identify studies reporting psychometric properties of the Tampa Scale for Kinesiophobia for Temporomandibular Disorders (TSK-TMD). The final searches were conducted in March 2026. These databases were selected because, together, they provide broad coverage of biomedical, dental, multidisciplinary, and citation-indexed literature relevant to temporomandibular disorders and psychometric validation. Embase, PsycINFO, and grey literature sources were not systematically searched.

Database-specific search strategies were developed using combinations of terms related to kinesiophobia, temporomandibular disorders, and psychometric measurement properties, with syntax adapted to the requirements of each database. The complete search strategies, search fields, search dates, and filters applied are presented in Supplementary Table S1.

Searches were limited to publications in English. Where available, the document type was restricted to journal articles. All retrieved records were imported into Zotero, where duplicate records were identified and removed before the screening process. Titles and abstracts were screened according to the predefined eligibility criteria. Full-text articles considered potentially eligible were subsequently assessed for inclusion. Additionally, the reference lists of the included studies were manually screened to identify any further relevant publications.

### 2.6. Study Selection

All records identified through the database searches were imported into reference management software, and duplicate records were removed. The remaining records were screened according to the eligibility criteria. Titles and abstracts were first assessed to exclude clearly irrelevant records. The full texts of potentially eligible articles were then retrieved and evaluated for inclusion.

The study selection process was conducted by M.L. Subsequently, D.C.Z. performed a quality-control review of the screening decisions and verified the eligibility of the included studies. However, not all records were screened independently in duplicate. In cases of uncertainty, eligibility decisions were discussed with M.S.P. and D.C.Z. until consensus was reached.

The selection process was documented and presented using a PRISMA flow diagram adapted for scoping reviews. Reasons for exclusion were recorded where applicable.

### 2.7. Data Charting Process

Data from the included studies were charted by M.L. using a predefined data extraction form. D.C.Z. subsequently performed a quality-control check for consistency, accuracy, and completeness; data extraction was not conducted independently in duplicate. Any uncertainties were discussed with M.S.P. and D.C.Z. until consensus was reached.

The extracted information included:

- author and year of publication;
- country and language version of the TSK-TMD;
- study design;
- sample size;
- participant characteristics;
- clinical or non-clinical population;
- diagnostic criteria for TMD, where reported;
- version of the TSK-TMD evaluated;
- measurement properties assessed;
- main psychometric findings;
- comparator instruments used;
- main conclusions reported by the authors.

The predefined measurement properties considered during data charting were internal consistency, test–retest reliability, structural validity, construct validity (including convergent validity), measurement error, responsiveness, interpretability, minimal important change, and cross-cultural measurement invariance.

### 2.8. Methodological Quality Appraisal

Although critical appraisal is not mandatory for scoping reviews, the methodological quality of the included measurement-property studies was assessed using the COSMIN Risk of Bias checklist for PROMs, version July 2018 [23]. For each study, only the COSMIN boxes corresponding to the measurement properties investigated were completed. Applicable checklist items were rated as very good, adequate, doubtful, or inadequate. Items that were not applicable to the statistical method used were marked as not applicable, while measurement properties that were not investigated were classified as not assessed.

In accordance with COSMIN methodology [24,25], the overall risk-of-bias rating for each measurement property was determined using the “worst score counts” principle, whereby the lowest rating assigned to an applicable item determined the final rating for that COSMIN box. The appraisal was initially performed by M.L. and independently reviewed by D.C.Z. Disagreements or uncertainties were resolved through discussion and consensus with M.S.P.

Final ratings are summarized in Supplementary Table S2, whereas the complete item-by-item appraisal, including the supporting evidence and justification for each rating, is provided in Supplementary Table S3.

### 2.9. Synthesis of Results

The results were synthesized narratively and presented in tables to map the available evidence on the TSK-TMD. Studies were grouped according to language version, cultural context, population, and measurement properties assessed.

The synthesis focused on identifying:

- which versions of the TSK-TMD have been evaluated;
- which psychometric properties have been investigated;
- the range of reported reliability and validity estimates;
- methodological strengths and limitations of the available studies;
- gaps in the current literature requiring further investigation.

No meta-analysis was performed because of the small number of included studies and heterogeneity in populations, language versions, comparator instruments, and psychometric methods. Evidence gaps were identified based on the predefined measurement properties included in the data-charting framework. A gap was recorded when a measurement property was not reported or was insufficiently evaluated in the included studies.

## 3. Results

### 3.1. Study Selection

The database searches identified 117 records: 51 from PubMed, 19 from Scopus, and 47 from the Web of Science Core Collection. After removing 56 duplicate records, 61 unique records were screened based on title and abstract. Of these, 56 records were excluded because they did not evaluate the TSK-TMD, investigated a different outcome measure, were review articles, or did not report measurement properties of the instrument.

**Figure 1:**
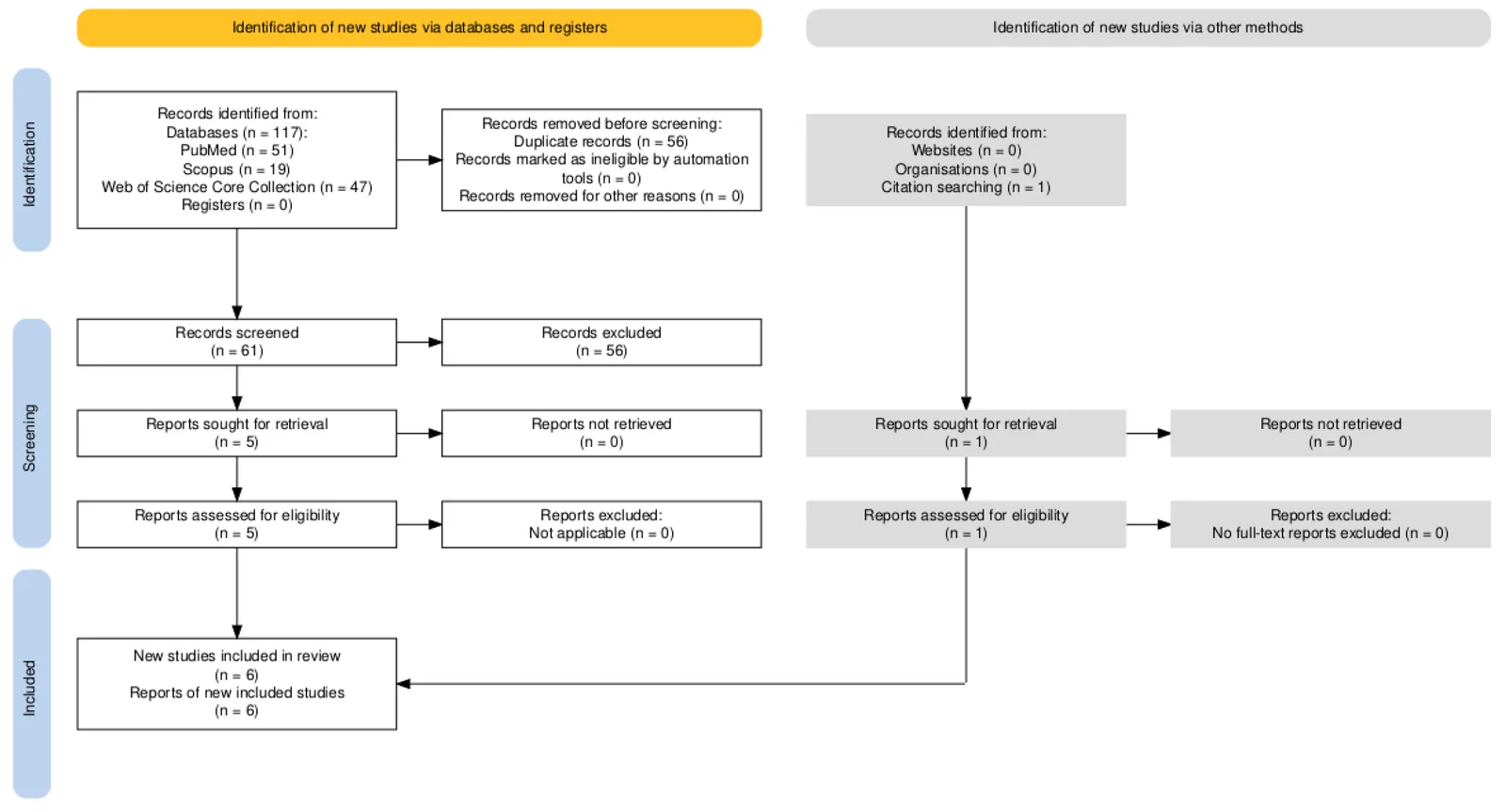
PRISMA 2020 flow diagram of the study selection process.

Five full-text articles identified through database searching were assessed for eligibility. In addition, one relevant article was identified through manual screening of the reference lists of the included studies. All six studies met the eligibility criteria and were included in the scoping review. The study selection process is presented in Figure 1.

### 3.2. Characteristics of Included Studies

Six studies evaluating the TSK-TMD or one of its translated and culturally adapted versions were included in this scoping review [15,16,17,18,19,20]. The studies were published between 2010 and 2024 and represented several cultural and linguistic contexts, including the original Dutch version and adaptations into Chinese, Korean, Spanish, Brazilian Portuguese, and Turkish.

Sample sizes ranged from 90 to 301 participants. Most studies included individuals diagnosed with temporomandibular disorders, while one study evaluated the instrument in symptom-free individuals from the general population. The included studies investigated different measurement properties of the TSK-TMD, including internal consistency, test–retest reliability, structural validity, and construct validity (including convergent validity).

The original instrument was developed by Visscher et al. in the Netherlands using a sample of patients with temporomandibular disorders. Subsequent studies focused mainly on translation, cross-cultural adaptation, and psychometric evaluation of the instrument in different populations. A summary of the characteristics of the included studies is presented in Table 1.

### 3.3. Mapping of Measurement Properties

The included studies evaluated internal consistency, test–retest reliability, structural validity, and construct validity, particularly convergent validity, across the available TSK-TMD versions. Internal consistency and test–retest reliability were reported in all six studies [15,16,17,18,19,20], whereas neither structural validity nor construct validity was assessed in the Korean adaptation [18]. Additional properties, including floor and ceiling effects and discriminant validity, were reported mainly for the Spanish version [19]. Measurement error, responsiveness, minimal important change, interpretability, and cross-cultural measurement invariance remained insufficiently investigated. A comprehensive summary of the psychometric findings and evidence gaps by TSK-TMD version is presented in Table 2.

### 3.4. Internal Consistency

Internal consistency was reported in all included studies, with Cronbach’s alpha coefficients ranging from 0.78 to 0.919 [15,16,17,18,19,20]. The findings indicated acceptable to excellent internal consistency across the available TSK-TMD versions, although interpretation should be considered together with the dimensional structure of each version. Version- specific estimates are presented in Table 2.

### 3.5. Test–Retest Reliability

Test–retest reliability was assessed in all included studies using intraclass correlation coefficients, which ranged from 0.73 to 0.951 [15,16,17,18,19,20]. The findings indicated acceptable to excellent temporal stability across the available studies, although retest intervals, sample characteristics, and participant stability criteria varied across studies. Version-specific estimates are presented in Table 2.

### 3.6. Structural Validity

Structural validity was evaluated in most of the included studies using exploratory factor analysis, confirmatory factor analysis, or both. Overall, the findings supported a two-factor structure corresponding to activity avoidance and somatic focus.

Confirmatory evidence was available for several adaptations, whereas the Chinese study used an exploratory approach and structural validity was not clearly assessed in the Korean version [15,16,17,18,19,20]. Further studies using robust confirmatory methods and measurement invariance testing are still needed. Version-specific findings are summarized in Table 2.

### 3.7. Construct Validity, Including Convergent Validity

Construct validity, particularly convergent validity, was assessed through associations between TSK-TMD scores and theoretically related constructs, including pain catastrophizing, pain intensity, disability, and psychological or functional measures. Overall, the included studies reported positive or moderate associations, although the magnitude of these relationships varied according to the study population, comparator instruments, cultural context, and clinical characteristics [15,16,17,18,19,20]. Version-specific findings are summarized in Table 2.

### 3.8. COSMIN Risk of Bias Appraisal

Methodological quality varied across studies and measurement properties. Structural validity was rated very good for the original Dutch, Brazilian Portuguese, and Turkish studies, adequate for the Chinese study, and doubtful for the Spanish study. Structural validity was not assessed in the Korean study.

Internal consistency was rated very good in the original Dutch, Chinese, Spanish, and Brazilian Portuguese studies, adequate in the Korean study, and inadequate in the Turkish study. The inadequate rating for the Turkish study resulted from reporting Cronbach’s alpha only for the total score despite support for a two-subscale structure.

Reliability was rated adequate for the original Dutch, Korean, Spanish, and Brazilian Portuguese studies, and doubtful for the Chinese and Turkish studies. The lower ratings primarily reflected insufficient information regarding participant stability, similarity of test conditions, the ICC model, or the appropriateness of the retest interval.

Measurement error was formally evaluated in the Spanish and Brazilian Portuguese studies and was rated adequate in both. It was not assessed in the remaining studies. Hypothesis testing for construct validity was rated very good for the original Dutch, Spanish, and Brazilian Portuguese studies, adequate for the Turkish study, and inadequate for the Chinese study. It was not assessed in the Korean study.

Final ratings for each study are presented in Table 3 and summarized in Supplementary Table S2. Detailed item-by-item ratings and justifications are provided in Supplementary Table S3.

## 4. Discussion

### 4.1. Summary of Evidence

This scoping review mapped the available evidence on the psychometric properties and cross-cultural adaptations of the Tampa Scale for Kinesiophobia for Temporomandibular Disorders (TSK-TMD). Six validation studies were identified, including the original Dutch version and adaptations developed in Chinese, Korean, Spanish, Brazilian Portuguese, and Turkish populations [15,16,17,18,19,20]. Most studies examined internal consistency and test–retest reliability, whereas structural validity and construct validity (including convergent validity) were evaluated less consistently.

Overall, the available findings suggest that the TSK-TMD is a promising condition-specific patient-reported outcome measure for assessing fear of movement in individuals with temporomandibular disorders. However, the evidence base remains limited in size and methodologically heterogeneous. Important measurement properties, including measurement error, responsiveness, minimal important change, interpretability, and cross-cultural measurement invariance, remain insufficiently investigated across the available language versions. Although the reported psychometric estimates were generally favorable, the methodological quality of the supporting studies varied across measurement properties. Therefore, favorable numerical findings should not automatically be interpreted as evidence of low risk of bias. Several measurement-property studies received adequate, doubtful, or inadequate COSMIN Risk of Bias ratings, mainly because of limitations in the reporting or design of reliability, internal-consistency, structural-validity, and construct-validity analyses.

### 4.2. Interpretation of Psychometric Findings

Internal consistency was generally supported across the available language versions. Nevertheless, internal consistency should be interpreted together with structural validity, because Cronbach’s alpha is influenced by the dimensionality of the instrument and cannot, by itself, establish that all items measure a single coherent construct. The generally favorable internal consistency findings are therefore encouraging, but their interpretation depends on whether the proposed factor structure is adequately supported in each population. However, favorable numerical estimates did not always correspond to high methodological quality. For example, the Turkish study reported a high total-score Cronbach’s alpha, but its internal-consistency risk-of-bias rating was inadequate because separate estimates were not provided for the two identified subscales.

Test–retest reliability was also consistently supported, suggesting that the TSK-TMD may provide stable scores when no relevant clinical change is expected. However, direct comparisons among studies should be made cautiously because retest intervals, participant characteristics, sample sizes, and criteria used to establish clinical stability differed across validation studies. These methodological differences may have contributed to variability in the reported reliability estimates. Although the reported ICC values were generally acceptable to excellent, the corresponding COSMIN Risk of Bias ratings were no higher than adequate and were doubtful in two studies. These lower methodological ratings mainly reflected insufficient documentation of participant stability, retest conditions, the appropriateness of the retest interval, or the ICC model. Thus, a high ICC value should be interpreted as a favorable numerical result, but not necessarily as evidence of high methodological quality.

Structural validity was investigated in most, but not all, included studies. The available evidence generally supported a two-factor structure corresponding to activity avoidance and somatic focus, which is consistent with the conceptual basis of kinesiophobia and the fear-avoidance model [9,10]. However, the strength of this evidence differed according to the analytical methods used. Studies applying confirmatory factor analysis and reporting several model-fit indices provided stronger support for the proposed structure, whereas studies relying mainly on exploratory factor analysis offered more preliminary evidence. Moreover, structural validity was not clearly evaluated in the Korean adaptation, and measurement invariance across language versions was not examined.

Differences among the validation studies should also be interpreted in relation to their study populations and cross-cultural adaptation procedures. The original Dutch study and most translated versions were evaluated in clinical TMD populations, whereas the Korean adaptation was studied in symptom-free individuals. This difference limits direct comparison with patient-based validation studies and may partly explain why a narrower range of measurement properties was investigated in that study. Variation in diagnostic criteria, clinical severity, sample composition, translation procedures, and cultural context may also have influenced the reported psychometric findings.

Construct validity, including convergent validity, was generally supported through associations between TSK-TMD scores and theoretically related variables, such as pain catastrophizing, pain intensity, disability, depression, jaw functional limitation, and craniofacial pain-related measures. These relationships are consistent with the biopsychosocial and fear-avoidance models of persistent pain. Nevertheless, the magnitude of the associations varied across studies, and the comparator instruments and hypotheses were not uniform. Consequently, the available findings support the expected relationship between kinesiophobia and related psychological and functional constructs, but they do not yet establish equivalent construct validity across all language versions.

Taken together, the findings should be regarded as favorable but preliminary. The small number of validation studies, the heterogeneity of the populations and psychometric methods, and the absence of formal cross-cultural measurement invariance testing prevent firm conclusions regarding the equivalence of the TSK-TMD across languages and cultural contexts.

### 4.3. Gaps in the Literature

An important finding of this scoping review is that several measurement properties remain insufficiently investigated. Measurement error was evaluated in only two of the included studies and therefore remains insufficiently investigated across the available TSK-TMD versions. This limits the ability to determine whether observed differences in TSK-TMD scores reflect true change or measurement variability.

Responsiveness was also not consistently assessed. This is a relevant limitation because the TSK-TMD may be used in clinical follow-up or intervention studies to monitor changes in fear of movement after treatment. Without evidence of responsiveness, it remains unclear whether the instrument can detect clinically meaningful change over time.

Minimal important change and interpretability were also not established. These properties are important for clinical decision-making because they help clinicians and researchers understand what degree of change in score is meaningful for patients. Similarly, cross-cultural measurement invariance was not reported, which limits confidence when comparing TSK-TMD scores across different language versions and cultural settings.

### 4.4. Clinical and Research Implications

The findings of this review suggest that the TSK-TMD may be a useful condition-specific patient-reported outcome measure for assessing fear of movement in individuals with temporomandibular disorders. Since TMD is influenced by biological, psychological, and social factors, assessing kinesiophobia may provide clinically relevant information beyond pain intensity or mandibular impairment alone [1,2,5].

In clinical practice, the TSK-TMD may help identify patients who avoid mandibular movement because of fear of pain or re-injury. This information may support individualized treatment planning, including patient education, reassurance regarding safe movement, therapeutic exercise, and multidisciplinary rehabilitation strategies.

For research, the TSK-TMD appears useful for observational studies, validation studies, and clinical trials investigating psychosocial factors in TMD. However, researchers should ensure that the version used has been adequately validated for the target language, population, and context. Direct comparisons across studies and countries should be made cautiously until stronger evidence on measurement invariance is available.

### 4.5. Strengths and Limitations

The strength of this review is its specific focus on the TSK-TMD as a condition-specific instrument for assessing kinesiophobia in temporomandibular disorders. By mapping the available literature across different cultural adaptations, this review provides an overview of the current state of psychometric evidence and highlights measurement properties that require further investigation.

Another strength is the use of a scoping review approach guided by recognized methodological and reporting frameworks. Searches were conducted in three major databases, PubMed, Scopus, and the Web of Science Core Collection, and were supplemented by manual reference screening. The inclusion of a descriptive COSMIN appraisal also provides additional information regarding the methodological quality of the available psychometric studies.

Several limitations should be acknowledged. First, a protocol was not prospectively registered for this scoping review. Although protocol registration is not mandatory for scoping reviews, the absence of a registered protocol may have reduced transparency regarding methodological decisions made during the review process. Second, although PubMed, Scopus, and the Web of Science Core Collection were searched and the reference lists of the included studies were manually screened, Embase and PsycINFO were not included, and grey literature was not systematically searched. Consequently, relevant validation studies indexed exclusively in specialized databases or available outside peer-reviewed journals may have been missed.

Third, only English-language full-text articles were considered eligible, which may have led to the exclusion of relevant validation studies published in other languages.

Fourth, study selection and data charting were primarily performed by one reviewer and subsequently quality-checked by a second reviewer rather than conducted independently in duplicate, which may have increased the risk of selection or data-extraction errors.

Fifth, the number of studies included was small. Although six studies were identified, not all measurement properties were assessed consistently across all versions. One study evaluated the instrument in symptom-free individuals rather than a clinical TMD population, which may limit the applicability of those findings to patients with temporomandibular disorders.

Finally, although a formal item-by-item COSMIN Risk of Bias appraisal was performed, the review did not apply the full COSMIN systematic-review methodology for grading the certainty of the overall body of evidence. The ratings should therefore be interpreted as assessments of the methodological quality of individual studies and measurement properties, rather than as certainty ratings for the complete evidence base.

### 4.6. Future Research

Future studies should evaluate measurement properties that remain insufficiently investigated, particularly measurement error, responsiveness, minimal important change, interpretability, and cross-cultural measurement invariance. These properties are essential for determining whether the TSK-TMD can be used to monitor clinical change and compare scores across different cultural contexts.

Further validation studies should include larger and more diverse clinical samples, including patients with different TMD subtypes, pain durations, and psychosocial profiles. Future studies should also clearly report diagnostic criteria, retest intervals, participant stability criteria, comparator instruments, and predefined hypotheses for construct validity.

Research is also needed to investigate the predictive validity of the TSK-TMD. Specifically, future studies should examine whether baseline fear of movement predicts treatment adherence, pain persistence, disability, or response to rehabilitation interventions. Such evidence would strengthen the clinical utility of the instrument and clarify its role in biopsychosocial assessment and treatment planning.

## 5. Conclusions

This scoping review mapped the available evidence on the psychometric properties and cross-cultural adaptations of the Tampa Scale for Kinesiophobia for Temporomandibular Disorders. The findings indicate that internal consistency and test–retest reliability are the most consistently investigated measurement properties, with generally acceptable to excellent results across the included studies. Structural validity and construct validity (including convergent validity) were also supported in several language adaptations, although methodological approaches varied.

Despite these favorable findings, several important gaps remain. Measurement error, responsiveness, minimal important change, interpretability, and cross-cultural measurement invariance were not consistently reported. Future studies should address these properties to support the use of the TSK-TMD in clinical follow-up, treatment evaluation, and cross-cultural comparisons.

Overall, the TSK-TMD appears to be a promising condition-specific instrument for assessing fear of movement in individuals with temporomandibular disorders, but further psychometric research is needed to strengthen its evidence base and clarify its clinical and research utility across diverse populations.

## Figures and Tables

**Table 1:** Characteristics of included studies.

Author, Year	Country/Language Version	Sample Size	Population	TSK-TMD Version	Main Purpose	Measurement Properties Assessed
Visscher et al., 2010 [15]	Netherlands/Dutch	301	Patients with TMD	Original TSK-TMD	Development and psychometric evaluation of the TSK-TMD	IC, TRR, SV, CV
He et al., 2016 [16]	China/Chinese	160	Patients with painful TMD	Chinese TSK-TMD	Translation, cross-cultural adaptation, and validation	IC, TRR, SV, CV
Aguiar et al., 2017 [17]	Brazil/Brazilian Portuguese	100	Female patients with chronic TMD	TSK/TMD—Br	Cross-cultural adaptation and psychometric evaluation	IC, TRR, ME, SV, CV
Park et al., 2018 [18]	Korea/Korean	90	Symptom-free individuals	Korean TSK-TMD	Translation and reliability assessment	IC, TRR
La Touche et al., 2020 [19]	Spain/Spanish	110	Patients with TMD	TSK-TMD—S	Translation, cross-cultural adaptation, and psychometric evaluation	IC, TRR, ME, CV, SV, F/CE, DV
Küçük et al., 2024 [20]	Turkey/Turkish	111	Patients with temporomandibular joint disorder	Turkish TSK-TMD	Translation and evaluation of reliability and validity	IC, TRR, SV, CV

Abbreviations: IC—Internal consistency; TRR—test–retest reliability; SV—structural validity; CV—convergent validity; F/CE—floor/ceiling effects; DV—discriminant validity; ME—measurement error.

**Table 2:** Comprehensive Summary of Psychometric Findings and Evidence Gaps by TSK-TMD Version.

TSK-TMD Version	Internal Consistency and Reliability	Structural Validity	Construct Validity (Including Convergent Validity)	Additional Properties and Evidence Gaps
Original Dutch version [15]	Cronbach’s α = 0.83 ICC = 0.73	Two-factor structure supported; factors corresponded to activity avoidance and somatic focus.	Positive association with pain catastrophizing; higher fear of movement was associated with functional TMD-related problems.	ME, responsiveness, MIC, interpretability, and cross-cultural measurement invariance were not reported.
Chinese version [16]	Cronbach’s α = 0.919 ICC = 0.797	Two-factor solution identified through exploratory factor analysis.	Moderate correlations with related pain-related and oral-health-related measures.	Further confirmatory factor analysis and measurement invariance testing are needed; other advanced measurement properties were not reported.
Korean version [18]	Cronbach’s α = 0.858 ICC = 0.752	Not reported.	Not reported	Evidence was mainly limited to internal consistency and test–retest reliability; structural and construct validity require further evaluation
Spanish version [19]	Cronbach’s α = 0.843 ICC = 0.904	Two-factor structure supported through factor analysis.	Moderate associations with craniofacial pain and disability, pain intensity, pain catastrophizing, and mouth-opening measures.	No floor or ceiling effects were identified, and discriminant validity was assessed. Measurement error was evaluated in this study, but further confirmation required; responsiveness, MIC and measurement invariance were not evaluated.
Brazilian Portuguese version [17]	Cronbach’s α = 0.78 ICC > 0.75	Two-domain, 12-item structure supported.	Moderate correlations with pain catastrophizing, depression, and jaw functional limitation.	Further assessment of measurement error, responsiveness, interpretability, minimal important change, and measurement invariance is needed.
Turkish version [20]	Cronbach’s α = 0.876 ICC = 0.951	Factor structure reported as acceptable.	Moderate correlation with pain catastrophizing.	Further confirmatory assessment and evaluation of measurement error, responsiveness, interpretability, minimal important change, and measurement invariance are needed.

Abbreviations: ICC, intraclass correlation coefficient; ME, measurement error; MIC, minimal important change; TSK-TMD—Tampa Scale for Kinesiophobia for Temporomandibular Disorders. Note: values are reported as presented in the included studies. Where measurement property was not reported, this was indicated accordingly. Evidence gaps were identified when predefined measurement properties were not reported or were insufficiently evaluated in the included studies.

**Table 3:** COSMIN Risk of Bias ratings of the included validation studies.

Study/Version	Structural Validity	Internal Consistency	Reliability	Measurement Error	Construct Validity
Dutch/original [15]	Very good	Very good	Adequate	Not assessed	Very good
Chinese [16]	Adequate	Very good	Doubtful	Not assessed	Inadequate
Korean [18]	Not assessed	Adequate	Adequate	Not assessed	Not assessed
Spanish [19]	Doubtful	Very good	Adequate	Adequate	Very good
Brazilian Portuguese [17]	Very good	Very good	Adequate	Adequate	Very good
Turkish [20]	Very good	Inadequate	Doubtful	Not assessed	Adequate

**Note:** Final ratings were determined using the COSMIN “worst score counts” principle. “Not assessed” indicates that the respective measurement property was not investigated in that study. The complete item-by-item appraisal and justification are provided in Supplementary Table S3.

## Data Availability

All data generated or analyzed during this review are included in this article and its Supplementary Materials. Further inquiries can be directed to the corresponding author.

## Supplementary Materials

The following supporting information can be downloaded at: https://www.mdpi.com/article/10.3390/dj14080508/s1, File S1: PRISMA-ScR checklist; Table S1. Complete search strategies used in literature search; Table S2. Summary of final COSMIN Risk of Bias ratings; Table S3. Detailed item-by-item COSMIN Risk of Bias appraisal.

## Abbreviations

The following abbreviations are used in this manuscript:

CFA	Confirmatory factor analysis
COSMIN	Consensus-based Standards for the selection of health Measurement INstruments
CV	Convergent validity
DC/TMD	Diagnostic Criteria for Temporomandibular Disorders
DV	Discriminant validity
EFA	Exploratory factor analysis
F/CE	Floor and ceiling effects
IC	Internal consistency
ICC	Intraclass correlation coefficient
JBI	Joanna Briggs Institute
MIC	Minimal important change
PCC	Population-Concept-Context
PRISMA-ScR	Preferred reporting Items for Systematic Review and Meta-Analyses extension for Scoping Review
SV	Structural validity
TMD	Temporomandibular Disorders
TRR	Test–retest reliability
TSK	Tampa Scale for Kinesiophobia
TSK-TMD	Tampa Scale for Kinesiophobia for Temporomandibular Disorders
