# Supplementary material for: Psychometric Evidence on the Tampa Scale for Kinesiophobia for Temporomandibular Disorders: A Scoping Review
Source: Dent J (Basel). 2026 Aug 11;14(8):508. doi: 10.3390/dj14080508 (PMC13510727; doi:10.3390/dj14080508)
Supplement: Supplementary file 1 [file dentistry-14-00508-s001.zip › Supplementary Table S1.pdf]

Supplementary Table S1. Complete search strategies used in the literature search.

Database	Search date	Search field(s)	Filters applied	Complete search strategy
**PubMed**	14 March 2026	Title/Abstract	English	("kinesiophobia"[Title/Abstract] OR "Tampa Scale for Kinesiophobia"[Title/Abstract] OR "TSK-TMD"[Title/Abstract] OR "TSK TMD"[Title/Abstract]) AND ("temporomandibular disorder*"[Title/Abstract] OR "temporomandibular dysfunction"[Title/Abstract] OR "TMD"[Title/Abstract] OR "orofacial pain"[Title/Abstract])
**Scopus**	21 March 2026	TITLE-ABS-KEY	English; Article	TITLE-ABS-KEY (("kinesiophobia" OR "Tampa Scale for Kinesiophobia" OR "TSK-TMD" OR "TSK TMD") AND ("temporomandibular disorder*" OR "temporomandibular dysfunction" OR "TMD" OR "orofacial pain") AND ("psychometric*" OR "measurement propert*" OR valid* OR reliab* OR validation))
**Web of Science Core Collection**	31 March 2026	Topic (TS)	English; Article	("kinesiophobia" OR "Tampa Scale for Kinesiophobia" OR "TSK-TMD" OR "TSK TMD") AND ("temporomandibular disorder*" OR "temporomandibular dysfunction" OR "TMD" OR "orofacial pain") AND ("psychometric*" OR "measurement propert*" OR valid* OR reliab* OR validation)

Note

Duplicate records were identified and removed using Zotero. In addition, the reference lists of all included studies were manually screened to identify potentially eligible studies. One additional study was identified through backward reference searching.
