# Supplementary material for: Psychometric Evidence on the Tampa Scale for Kinesiophobia for Temporomandibular Disorders: A Scoping Review
Source: Dent J (Basel). 2026 Aug 11;14(8):508. doi: 10.3390/dj14080508 (PMC13510727; doi:10.3390/dj14080508)
Supplement: Supplementary file 1 [file dentistry-14-00508-s001.zip › Supplementary Table S2.pdf]

## Supplementary Table S2. Summary of final COSMIN Risk of Bias ratings

Final methodological ratings by study and measurement property. Detailed item-level evidence and justifications are presented in Supplementary Table S3.

| Study | Structural validity | Internal consistency | Reliability | Measurement error | Construct validity | Main rationale |
|---|---|---|---|---|---|---|
| Visscher et al. 2010 [15] | Very good | Very good | Adequate | Not assessed | Very good | CFA with adequate sample; alpha by subscale; reliability downgraded because stability/test conditions were assumed rather than demonstrated. |
| He et al. 2016 [16] | Adequate | Very good | Doubtful | Not assessed | Inadequate | EFA (not CFA) determines structural rating; reliability limited by unclear test conditions and unspecified ICC model; single global oral-health item had no demonstrated measurement properties. |
| Park et al. 2018 [18] | Not assessed | Adequate | Adequate | Not assessed | Not assessed | No factor analysis or formal construct-validity study. Internal consistency was calculated by subscale, but dimensionality was not tested in the Korean sample. |
| La Touche et al. 2020 [19] | Doubtful | Very good | Adequate | Adequate | Very good | EFA and CFA were performed in the same sample after item reduction, creating possible overfitting; ICC model was not reported; SEM/MDC were calculated. |
| Aguiar et al. 2017 [17] | Very good | Very good | Adequate | Adequate | Very good | Pre-specified CFA with n=100; ICC model reported. Stability and identical test conditions were assumed rather than explicitly demonstrated. |
| Küçük et al. 2024 [20] | Very good | Inadequate | Doubtful | Not assessed | Adequate | CFA and sample size were adequate. Cronbach alpha was reported only for the total score despite a two-subscale structure. Retest interval was very short and stability/test conditions were unclear. |

**Note.** Risk-of-bias ratings evaluate the methodological quality of each measurement-property study and are distinct from ratings of s sufficiency of the numerical results (+, −, or ?). Final ratings were determined using COSMIN "worst score counts" principle. "Not assessed" indicates that the respective measurement property was not investigated. Detailed item-level evidence and justifications are presented in Supplementary Table 3.
