# Supplementary material for: Psychometric Evidence on the Tampa Scale for Kinesiophobia for Temporomandibular Disorders: A Scoping Review
Source: Dent J (Basel). 2026 Aug 11;14(8):508. doi: 10.3390/dj14080508 (PMC13510727; doi:10.3390/dj14080508)
Supplement: Supplementary file 1 [file dentistry-14-00508-s001.zip › Supplementary Table S3.pdf]

Supplementary Table S3. Detailed item-by-item COSMIN Risk of Bias appraisal

Each subsection reports the evidence extracted from the original article, the item-level rating, and the justification. The final property rating is shown in the subsection heading and follows the worst-score-counts rule.

Visscher et al. 2010 [15]

Final ratings: Structural validity — Very good; Internal consistency — Very good; Reliability — Adequate; Measurement error — Not assessed; Construct validity — Very good.

Box 3 — Structural validity — Final rating: Very good

Item	COSMIN criterion	Evidence from article	Item rating	Justification	Source location
3.1	CTT factor analysis performed	Confirmatory factor analysis compared five candidate models.	Very good	CFA satisfies the highest COSMIN category.	Dutch PDF pp. 4–5
3.3	Adequate sample size	n=301; analyses concerned 18 or fewer items.	Very good	n exceeded 7 × number of items and was ≥100.	Dutch PDF pp. 4–5
3.4	Other important flaws	Ordinal items analysed using polychoric correlations, robust correction and a justified estimation approach; multiple fit indices reported.	Very good	No major additional flaw identified.	Dutch PDF pp. 4–6

Box 4 — Internal consistency — Final rating: Very good

Item	COSMIN criterion	Evidence from article	Item rating	Justification	Source location
4.1	Statistic for each unidimensional scale/subscale	Cronbach alpha reported for Activity Avoidance and Somatic Focus after CFA, and for total score.	Very good	Alpha was calculated for the identified subscales.	Dutch PDF pp. 5–6
4.2	Cronbach alpha or omega calculated	Cronbach alpha was calculated.	Very good	Appropriate statistic for continuous scores.	Dutch PDF pp. 5–6
4.5	Other important flaws	No material design/statistical flaw identified.	Very good	No downgrade required.	Dutch PDF pp. 5–6

Box 6 — Reliability — Final rating: Adequate

Item	COSMIN criterion	Evidence from article	Item rating	Justification	Source location
6.1	Patients stable in interim	Second assessment occurred before the first clinic visit; no explicit stability assessment was reported.	Adequate	Stability is plausible but not demonstrated.	Dutch PDF p. 4
6.2	Time interval appropriate	Retest was within four weeks.	Very good	Reasonable interval for a relatively stable construct and sufficient to reduce recall.	Dutch PDF p. 4
6.3	Test conditions similar	Same PROM was repeated, but administration conditions were not explicitly compared.	Adequate	Similarity is assumable, not evidenced.	Dutch PDF p. 4
6.4	ICC and model/formula described	Two-way mixed-effects, absolute-agreement, single-measure ICC was specified.	Very good	Fully specified ICC model.	Dutch PDF p. 5

Item	COSMIN criterion	Evidence from article	Item rating	Justification	Source location
6.8	Other important flaws	No other important flaw identified.	Very good	No downgrade required.	Dutch PDF pp. 4–6

Box 9a — Construct validity — Final rating: Very good

Item	COSMIN criterion	Evidence from article	Item rating	Justification	Source location
9a.1	Comparator construct clear	Catastrophizing scale and its theoretical relationship to fear avoidance were described.	Very good	Comparator construct was explicit.	Dutch PDF p. 4
9a.2	Comparator measurement properties sufficient	Internal consistency, test–retest reliability and construct validity of the catastrophizing scale were reported/cited.	Very good	Sufficient evidence for the comparator was provided.	Dutch PDF p. 4
9a.3	Appropriate statistical method	Pearson correlation was used.	Very good	Appropriate for the planned convergent comparison.	Dutch PDF pp. 5–6
9a.4	Other important flaws	No important additional flaw identified.	Very good	No downgrade required.	Dutch PDF pp. 4–6

He et al. 2016 [16]

Final ratings: Structural validity — Adequate; Internal consistency — Very good; Reliability — Doubtful; Measurement error — Not assessed; Construct validity — Inadequate.

Box 3 — Structural validity — Final rating: Adequate

Item	COSMIN criterion	Evidence from article	Item rating	Justification	Source location
3.1	CTT factor analysis performed	Exploratory factor analysis with varimax rotation.	Adequate	EFA corresponds to COSMIN 'adequate'; CFA would be 'very good'.	Chinese PDF pp. 2–3
3.3	Adequate sample size	n=160 for 12 items.	Very good	Exceeds 7×12 and ≥100.	Chinese PDF pp. 2–3
3.4	Other important flaws	KMO and Bartlett tests, extraction and loading threshold were reported.	Very good	No major additional flaw identified.	Chinese PDF pp. 2–3

Box 4 — Internal consistency — Final rating: Very good

Item	COSMIN criterion	Evidence from article	Item rating	Justification	Source location
4.1	Statistic for each unidimensional scale/subscale	Alpha reported for total score and both EFA-derived subscales.	Very good	Subscale-specific estimates were provided after dimensionality analysis.	Chinese PDF pp. 3–4
4.2	Cronbach alpha or omega calculated	Cronbach alpha calculated.	Very good	Appropriate statistic.	Chinese PDF pp. 3–4
4.5	Other important flaws	No important flaw identified.	Very good	No downgrade required.	Chinese PDF pp. 3–4

Box 6 — Reliability — Final rating: Doubtful

Item	COSMIN criterion	Evidence from article	Item rating	Justification	Source location
6.1	Patients stable in interim	No treatment occurred during the two-week interval.	Adequate	Stability is plausible, but no direct stability criterion was measured.	Chinese PDF p. 3
6.2	Time interval appropriate	Two-week interval.	Very good	Appropriate interval.	Chinese PDF p. 3
6.3	Test conditions similar	Conditions at the two administrations were not described.	Doubtful	Similarity cannot be established from the report.	Chinese PDF p. 3
6.4	ICC and model/formula described	ICC reported, but model/formula not specified.	Adequate	COSMIN rates an unspecified ICC model as adequate.	Chinese PDF pp. 3–4
6.8	Other important flaws	Retest sample n=30; no other explicit flaw.	Very good	The 2018 Box 6 has no separate sample-size item.	Chinese PDF pp. 3–4

Box 9a — Construct validity — Final rating: Inadequate

Item	COSMIN criterion	Evidence from article	Item rating	Justification	Source location
9a.1	Comparator construct clear	Single global question rated temporomandibular-joint/oral health status.	Very good	The comparator construct was stated.	Chinese PDF p. 2
9a.2	Comparator measurement properties sufficient	No validity or reliability evidence was provided for the single global question.	Inadequate	COSMIN requires information supporting the comparator's measurement properties.	Chinese PDF pp. 2–3
9a.3	Appropriate statistical method	Correlation analysis was used.	Very good	Appropriate for convergent association.	Chinese PDF pp. 3–4
9a.4	Other important flaws	No additional major flaw beyond comparator quality.	Very good	No further downgrade.	Chinese PDF pp. 3–4

Park et al. 2018 [18]

Final ratings: Structural validity — Not assessed; Internal consistency — Adequate; Reliability — Adequate; Measurement error — Not assessed; Construct validity — Not assessed.

Box 4 — Internal consistency — Final rating: Adequate

Item	COSMIN criterion	Evidence from article	Item rating	Justification	Source location
4.1	Statistic for each unidimensional scale/subscale	Alpha reported for Activity Avoidance and Somatic Focus, but dimensionality was not evaluated in the Korean sample.	Adequate	It is unclear whether the translated subscales are unidimensional in this sample.	Korean PDF pp. 1, 4–5
4.2	Cronbach alpha or omega calculated	Cronbach alpha calculated.	Very good	Appropriate statistic.	Korean PDF pp. 1, 4–5
4.5	Other important flaws	Study population was symptom-free rather than the intended clinical TMD population.	Adequate	This is a relevant generalisability limitation but not a fatal statistical flaw.	Korean PDF pp. 1–2

Box 6 — Reliability — Final rating: Adequate

Item	COSMIN criterion	Evidence from article	Item rating	Justification	Source location
6.1	Patients stable in interim	Symptom-free participants; interval selected to avoid meaningful change.	Very good	The sample and rationale support stability.	Korean PDF pp. 3–4
6.2	Time interval appropriate	One or two weeks, explicitly justified.	Very good	Appropriate interval.	Korean PDF pp. 3–4

Item	COSMIN criterion	Evidence from article	Item rating	Justification	Source location
6.3	Test conditions similar	Same questionnaire repeated using a simple-blind procedure; environment details not fully reported.	Adequate	Conditions appear similar but were not fully documented.	Korean PDF pp. 3–4
6.4	ICC and model/formula described	ICC reported; model/formula not stated.	Adequate	Unspecified ICC model is adequate, not very good.	Korean PDF pp. 3–5
6.8	Other important flaws	Clinical target population not represented.	Adequate	Limits applicability, although temporal reliability was estimable.	Korean PDF pp. 1–2

La Touche et al. 2020 [19]

Final ratings: Structural validity — Doubtful; Internal consistency — Very good; Reliability — Adequate; Measurement error — Adequate; Construct validity — Very good.

Box 3 — Structural validity — Final rating: Doubtful

Item	COSMIN criterion	Evidence from article	Item rating	Justification	Source location
3.1	CTT factor analysis performed	EFA followed by CFA.	Very good	CFA was performed.	Spanish PDF pp. 4, 7–8
3.3	Adequate sample size	Approximately 110–120 participants for 12 initial items.	Very good	At least 7×12 and ≥100.	Spanish PDF pp. 3–4
3.4	Other important flaws	EFA-driven item reduction and CFA were conducted in the same sample, without independent validation.	Doubtful	Potential overfitting is an important methodological limitation.	Spanish PDF pp. 4, 7–8

Box 4 — Internal consistency — Final rating: Very good

Item	COSMIN criterion	Evidence from article	Item rating	Justification	Source location
4.1	Statistic for each unidimensional scale/subscale	Alpha reported for total and both CFA-derived subscales.	Very good	Subscale-specific estimates were reported.	Spanish PDF p. 8
4.2	Cronbach alpha or omega calculated	Cronbach alpha calculated.	Very good	Appropriate statistic.	Spanish PDF p. 8
4.5	Other important flaws	No other important flaw specific to alpha calculation.	Very good	No downgrade.	Spanish PDF p. 8

Box 6 — Reliability — Final rating: Adequate

Item	COSMIN criterion	Evidence from article	Item rating	Justification	Source location
6.1	Patients stable in interim	Chronic TMD sample; no explicit stability criterion or treatment restriction reported.	Adequate	Stability is plausible but not demonstrated.	Spanish PDF pp. 4, 8
6.2	Time interval appropriate	Seven to ten days; retest reported at ten days.	Very good	Appropriate interval.	Spanish PDF pp. 4, 8
6.3	Test conditions similar	Administration conditions were not explicitly compared.	Adequate	Similarity is assumable.	Spanish PDF pp. 4, 8
6.4	ICC and model/formula described	ICC reported, but the model/formula was not specified.	Adequate	Unspecified ICC model.	Spanish PDF pp. 4, 8
6.8	Other important flaws	No additional important flaw identified.	Very good	No further downgrade.	Spanish PDF pp. 4, 8

Box 7 — Measurement error — Final rating: Adequate

Item	COSMIN criterion	Evidence from article	Item rating	Justification	Source location
7.1	Patients stable in interim	Same retest design as reliability; stability assumed.	Adequate	No explicit stability assessment.	Spanish PDF pp. 4, 8
7.2	Time interval appropriate	Seven to ten days.	Very good	Appropriate interval.	Spanish PDF pp. 4, 8
7.3	Test conditions similar	Not explicitly documented.	Adequate	Similarity assumed.	Spanish PDF pp. 4, 8
7.4	SEM, SDC/MDC or LoA calculated	SEM and MDC90/MDC95 calculated.	Very good	Appropriate measurement-error indices.	Spanish PDF pp. 4, 8
7.6	Other important flaws	SEM was derived from ICC; no other major flaw identified.	Very good	No further downgrade.	Spanish PDF pp. 4, 8

Box 9a — Construct validity — Final rating: Very good

Item	COSMIN criterion	Evidence from article	Item rating	Justification	Source location
9a.1	Comparator constructs clear	CF-PDI, VAS, general TSK, PCS and mouth opening were explicitly described.	Very good	Comparator constructs were clear.	Spanish PDF pp. 3–5
9a.2	Comparator measurement properties sufficient	Validated comparator instruments were used and referenced.	Very good	Adequate evidence for comparator quality.	Spanish PDF pp. 3–5
9a.3	Appropriate statistical method	Correlation analyses matched the hypotheses.	Very good	Appropriate methods.	Spanish PDF pp. 5, 8–9
9a.4	Other important flaws	No important flaw identified.	Very good	No downgrade.	Spanish PDF pp. 5, 8–9

Aguiar et al. 2017 [17]

Final ratings: Structural validity — Very good; Internal consistency — Very good; Reliability — Adequate; Measurement error — Adequate; Construct validity — Very good.

Box 3 — Structural validity — Final rating: Very good

Item	COSMIN criterion	Evidence from article	Item rating	Justification	Source location
3.1	CTT factor analysis performed	Pre-specified CFA compared three models.	Very good	CFA performed.	Brazilian PDF pp. 8, 10–11
3.3	Adequate sample size	n=100 for 12-item final model.	Very good	Meets ≥7×12 and ≥100.	Brazilian PDF pp. 4, 8
3.4	Other important flaws	Multiple fit indices reported; pre-specified models evaluated.	Very good	No major additional flaw identified.	Brazilian PDF pp. 8, 10–11

Box 4 — Internal consistency — Final rating: Very good

Item	COSMIN criterion	Evidence from article	Item rating	Justification	Source location

Item	COSMIN criterion	Evidence from article	Item rating	Justification	Source location
4.1	Statistic for each unidimensional scale/subscale	Alpha reported for both domains after CFA.	Very good	Subscale-specific estimates provided.	Brazilian PDF pp. 8, 10–11
4.2	Cronbach alpha or omega calculated	Cronbach alpha calculated.	Very good	Appropriate statistic.	Brazilian PDF pp. 8, 10–11
4.5	Other important flaws	No important flaw identified.	Very good	No downgrade.	Brazilian PDF pp. 8, 10–11

Box 6 — Reliability — Final rating: Adequate

Item	COSMIN criterion	Evidence from article	Item rating	Justification	Source location
6.1	Patients stable in interim	One-week interval chosen to limit clinical change; no direct stability check reported.	Adequate	Stability plausible but not demonstrated.	Brazilian PDF pp. 7–8, 11–12
6.2	Time interval appropriate	One week.	Very good	Appropriate interval.	Brazilian PDF pp. 7, 11–12
6.3	Test conditions similar	Same PROM repeated; conditions not explicitly compared.	Adequate	Similarity assumable.	Brazilian PDF pp. 7–8
6.4	ICC and model/formula described	Two-way mixed-effects, absolute-agreement ICC specified.	Very good	Fully specified ICC model.	Brazilian PDF p. 8
6.8	Other important flaws	No other important flaw identified.	Very good	No further downgrade.	Brazilian PDF pp. 7–8

Box 7 — Measurement error — Final rating: Adequate

Item	COSMIN criterion	Evidence from article	Item rating	Justification	Source location
7.1	Patients stable in interim	Stability assumed, not directly verified.	Adequate	Same limitation as reliability.	Brazilian PDF pp. 7–8, 11–12
7.2	Time interval appropriate	One week.	Very good	Appropriate.	Brazilian PDF pp. 7, 11–12
7.3	Test conditions similar	Not explicitly documented.	Adequate	Similarity assumed.	Brazilian PDF pp. 7–8
7.4	SEM, SDC or LoA calculated	SEM and SDC reported for domains and total score.	Very good	Appropriate indices calculated.	Brazilian PDF p. 12
7.6	Other important flaws	No other important flaw identified.	Very good	No further downgrade.	Brazilian PDF p. 12

Box 9a — Construct validity — Final rating: Very good

Item	COSMIN criterion	Evidence from article	Item rating	Justification	Source location
9a.1	Comparator constructs clear	Catastrophizing, depression and jaw-function instruments described.	Very good	Constructs clear.	Brazilian PDF pp. 5–8
9a.2	Comparator measurement properties sufficient	Brazilian versions and psychometric evidence were described/cited.	Very good	Comparator quality supported.	Brazilian PDF pp. 5–6
9a.3	Appropriate statistical method	Predefined hypotheses tested with Pearson correlations.	Very good	Appropriate analysis.	Brazilian PDF pp. 7–8
9a.4	Other important flaws	No important flaw identified.	Very good	No downgrade.	Brazilian PDF pp. 7–8, 10–11

Küçük et al. 2024 [20]

Final ratings: Structural validity — Very good; Internal consistency — Inadequate; Reliability — Doubtful; Measurement error — Not assessed; Construct validity — Adequate.

Box 3 — Structural validity — Final rating: Very good

Item	COSMIN criterion	Evidence from article	Item rating	Justification	Source location
3.1	CTT factor analysis performed	CFA of the original two-factor, 12-item structure.	Very good	CFA performed.	Turkish PDF pp. 4–5
3.3	Adequate sample size	n=111 for 12 items.	Very good	Meets ≥7×12 and ≥100.	Turkish PDF pp. 1, 4–5
3.4	Other important flaws	Multiple fit indices reported and acceptable; no major flaw identified.	Very good	No downgrade.	Turkish PDF pp. 4–5

Box 4 — Internal consistency — Final rating: Inadequate

Item	COSMIN criterion	Evidence from article	Item rating	Justification	Source location
4.1	Statistic for each unidimensional scale/subscale	Only total-score Cronbach alpha was reported despite a two-factor structure.	Inadequate	COSMIN requires an internal-consistency statistic for each unidimensional subscale.	Turkish PDF pp. 5–7
4.2	Cronbach alpha or omega calculated	Cronbach alpha calculated for total score.	Very good	Appropriate statistic, but not at the required subscale level.	Turkish PDF pp. 5–7
4.5	Other important flaws	No additional flaw beyond missing subscale estimates.	Very good	Overall remains inadequate by worst-score-counts.	Turkish PDF pp. 5–7

Box 6 — Reliability — Final rating: Doubtful

Item	COSMIN criterion	Evidence from article	Item rating	Justification	Source location
6.1	Patients stable in interim	No direct stability assessment or treatment restriction reported.	Doubtful	Stability is unclear.	Turkish PDF pp. 5–7
6.2	Time interval appropriate	Retest after 3–5 days.	Doubtful	Very short interval creates a meaningful recall risk.	Turkish PDF p. 7
6.3	Test conditions similar	Conditions were not described.	Doubtful	Cannot establish similarity.	Turkish PDF pp. 5–7
6.4	ICC and model/formula described	ICC reported, but model/formula not specified.	Adequate	Unspecified ICC model.	Turkish PDF pp. 5–7
6.8	Other important flaws	Only 40 patients were retested; no additional fatal flaw in the 2018 box.	Very good	No separate sample-size criterion in this box.	Turkish PDF p. 7

Box 9a — Construct validity — Final rating: Adequate

Item	COSMIN criterion	Evidence from article	Item rating	Justification	Source location
9a.1	Comparator construct clear	Pain Catastrophizing Scale construct described.	Very good	Comparator construct clear.	Turkish PDF pp. 3–5
9a.2	Comparator measurement properties sufficient	Turkish adaptation cited, but detailed psychometric evidence in a comparable TMD population was not presented.	Adequate	Comparator appears validated, but applicability to this population is not fully established.	Turkish PDF pp. 3–5

Item	COSMIN criterion	Evidence from article	Item rating	Justification	Source location
9a.3	Appropriate statistical method	Pearson correlation used.	Very good	Appropriate method.	Turkish PDF pp. 4–5
9a.4	Other important flaws	No additional important flaw identified.	Very good	No further downgrade.	Turkish PDF pp. 4–5

Rating legend

Rating	Interpretation	Effect on final box rating
Very good	Highest methodological standard met	Does not lower the final rating.
Adequate	Acceptable, with a methodological or reporting limitation	Final box cannot be higher than Adequate if this is the worst item.
Doubtful	Important uncertainty or methodological limitation	Final box becomes Doubtful if this is the worst item.
Inadequate	Method does not meet the COSMIN standard	Final box becomes Inadequate.
Not assessed	The property was not investigated	No poor rating is assigned.
